# Healthcare Providers’ Acceptability of Cannabis And Cannabidiol to Manage Parkinson’s Disease in France

**DOI:** 10.1016/j.curtheres.2026.100830

**Published:** 2026-04-03

**Authors:** Tangui Barré, Géraldine Cazorla, Vincent Di Beo, Carla Bianchi, Christelle Baunez, Patrizia Carrieri

**Affiliations:** 1 Aix-Marseille Univ, Inserm, IRD, SESSTIM, Sciences Economiques & Sociales de la Santé & Traitement de l’Information Médicale, ISSPAM, Marseille, France; 2 Institut de Neurosciences de la Timone (INT) UMR7289 CNRS & Aix-Marseille Université, Marseille, France

**Keywords:** Cannabidiol, Cannabis, Parkinson’s disease, Physicians

## Abstract

**Background:**

Some people living with Parkinson’s disease (PD) self-administer cannabis-based products to manage symptoms. However, using nonprescribed products can pose risks. In France, PD is not an eligible condition for prescribed cannabis-based products. Investigating healthcare providers’ perspectives on this topic is critical as PD might be considered for inclusion in future French medical cannabis programs.

**Objective:**

We aimed to explore healthcare providers’ acceptability and perceptions regarding the therapeutic use of cannabis and cannabidiol (CBD) for PD in France.

**Methods:**

An online cross-sectional survey was conducted among healthcare professionals managing patients with PD in France. Logistic regressions were performed to identify factors associated with their acceptability of cannabis and CBD use to manage PD.

**Results:**

The study population comprised 218 professionals, including 45 physicians. Acceptability levels were greater for CBD than for cannabis. Providers very concerned about cannabis dependence were less likely to agree with cannabis use to manage PD. Physicians were 87% and 90% less likely than other providers to agree with the use of cannabis and CBD, respectively, to manage PD. Among physicians, the primary barrier identified to the use of both substances was a lack of evidence to support their therapeutic effectiveness.

**Conclusions:**

Among a sample of healthcare providers in France, physicians showed lower acceptability towards cannabis and CBD for PD. CBD was generally more accepted than cannabis. If cannabis-based products were to be authorized for symptom alleviation in PD patients, physicians may require more robust scientific evidence and training before prescribing these treatments.

## Introduction

People with Parkinson’s disease (PD) often experience a reduced quality of life (QoL)^1^ due to motor symptoms (eg, bradykinesia, dyskinesia, tremor) and nonmotor symptoms (eg, depression, pain, sleep disorders).^2^, ^3^, ^4^ These symptoms impair QoL in most domains, especially physical function and mental health.^1^ The level of disability, resulting from these symptoms, also significantly shapes the overall impact on QoL.^2^ Treatment side effects can further compromise QoL.^3^^,^^5^^,^^6^ When standard treatments are insufficiently effective or have side effects, some patients turn to alternative remedies, such as cannabis and cannabidiol (CBD), whether medically prescribed or not.

Cannabis contains over 550 distinct chemical compounds, including more than 100 phytocannabinoids.^7^ Among them, tetrahydrocannabinol (THC) and CBD are the most abundant and extensively studied. THC exhibits psychoactive properties and is responsible for the “high” commonly associated with cannabis use. In contrast, CBD is nonintoxicating and well-tolerated. Both compounds, whether used individually or combined in plant extracts or pharmaceutical formulations, act on multiple biological targets and have shown putative therapeutic potential for various medical conditions.^8^

For instance, CBD is effective for individuals with epilepsy, and cannabis-based medicines may benefit those with multiple sclerosis, chronic pain, inflammatory bowel disease, and individuals in palliative care.^8^ Cannabinoids may exert therapeutic effects on neuroinflammation, oxidative stress, and excitotoxicity, key processes in PD pathogenesis.^9^^,^^10^ They may also alleviate symptoms such as levodopa-induced dyskinesia, anxiety, depression and sleep disorders.^9^ Systematic reviews and a meta-analysis have highlighted the potential of these products to relieve symptoms and improve QoL in people with PD.^11^, ^12^, ^13^ However, more robust evidence is still required, especially from large-scale and long-term randomized trials.^14^^,^^15^

Patients with PD who use cannabis-based products without medical supervision^16^, ^17^, ^18^, ^19^, ^20^, ^21^, ^22^ risk adverse effects, including drug-drug interactions with prescribed medications.^8^^,^^23^ Thus, healthcare provider support is critical—not only for safely integrating cannabis-based therapies into PD care where legally permitted, but also in contexts where such use remains prohibited. Providers’ acceptability—that is, the extent to which they deem cannabis-based products to be appropriate for PD—ultimately shapes patients’ ability to access these products safely. A judgmental or dismissive attitude toward therapeutic cannabis use may discourage patients from disclosing their use, thereby limiting providers' ability to mitigate associated risks. Healthcare providers' perspectives on this issue are likely to differ depending on whether they are physicians or other professionals, due to variations in training, professional responsibility, or therapeutic alliance.

Even in jurisdictions with legal medical cannabis programs, patients may encounter provider reluctance to prescribe or endorse its use, as observed in Canada^24^, ^25^, ^26^ and in Rønne et al’s systematic review.^27^ A survey of international neurologists, primarily from the US, revealed that while 95% had been asked to prescribe medical cannabis by patients, only 39% encouraged its use.^28^ This reluctance may reflect providers’ limited knowledge,^29^, ^30^, ^31^ fear of dependence,^32^ or their stigma towards people who use cannabis.^33^ Occupation-related differences in attitudes have also been documented. In the US, where most states permit the medical use of cannabis, a previous study found that neurologists held less favorable views toward therapeutic cannabis for PD than nurses did.^31^ While we may expect the same from countries without medical program, such data as still lacking. For instance, the acceptability of cannabis-based products for PD among French healthcare providers has yet to be documented. Consequently, even in supportive legal frameworks, healthcare providers’ attitudes may restrict patient access to medical cannabis. For instance, we showed that among French general practitioners, personal beliefs may overcome knowledge on cannabis as determinants to willingness to prescribe medical cannabis if it were available.^34^

In France, products containing THC above the threshold of 0.3% are illegal. This punitive cannabis policy comes along with cannabis-related stigma,^35^ which may possibly be also perpetuated by healthcare providers.^33^^,^^36^^,^^37^ The only exception to this restrictive regulatory environment is the national medical cannabis experimental project launched in 2021. This project aims to evaluate the feasibility of providing medical cannabis to individuals with severe chronic conditions who do not respond to conventional treatment.^38^ PD is not currently included among eligible conditions, despite patient-reported benefits^16^^,^^39^ (including in France^40^). Unlike THC, CBD is legal in France despite recent legal back-and-forth, but it remains prohibited in food products.^41^ About 10% of French adults use CBD,^42^^,^^43^ typically marketed as a wellness or complementary health product, and available in various forms including oils, creams, and THC-free cannabis flowers.

There is a lack of data on French healthcare providers’ attitudes towards cannabis-based products to manage PD. In a context where PD might be considered for inclusion in future French medical cannabis programs, understanding healthcare providers’ perspectives is crucial. We aimed to examine the acceptability, knowledge, and perceptions of healthcare providers regarding the therapeutic use of cannabis and CBD for PD in France, as well as barriers to endorsing such use. Specifically, factors associated with a higher acceptability will be identified.

## Material and Methods

### Study design and participants

A cross-sectional online survey was conducted from 16 September 2023, to 30 January 2024 utilizing the Voxco survey platform. Inclusion criteria were being a healthcare provider in France and actively managing patients with PD. The survey link was displayed through multiple channels. One was the France Parkinson association website (https://www.franceparkinson.fr/). Created in 1984 and a member of the European Parkinson’s Disease Association, France Parkinson is a national association recognized as being of public benefit, with 75 local committees throughout French territory.

Besides publicizing the generic survey link on France Parkinson’s website, invitations to participate in the survey were sent by email to approximately 5000 professionals in healthcare, social, and medico-social sectors via France Parkinson’s professional mailing list. Additionally, regional unions representing general practitioners, nurses, pharmacists, physiotherapists, and speech therapists were contacted by email or through their websites to disseminate the survey on their website or through the mailing list of their members. Similarly, national and local neurologist societies and neurology networks were also asked to forward the nonunique survey link to their members. Participants were encouraged to share the survey link to increase reach and participation. For data privacy reasons, IP addresses were not collected. Consequently, no check for duplicate responses was performed.

The survey was designed in accordance with the declaration of Helsinki, and was approved by the INSERM ethics committee (IRB00003888, CD/EB 23-045, 4 April 2023). Before accessing the survey questionnaire, participants had to provide informed consent by ticking a box. The survey questions were designed to prevent participant re-identification through data cross-referencing.

### Questionnaire and data collection

Eligibility was verified by asking participants: “Are you a healthcare provider actively managing patients with Parkinson’s disease?” Only those who answered “Yes” were granted access to the questionnaire.

Survey items were generated from literature review and adapted from a previous survey conducted in PD patients.^44^ The self-administered online questionnaire collected data on socio-demographic characteristics (age, gender, city size), and practice details (occupation, private or public sector, number of PD patients, years of experience with PD patients). Participants’ cannabinoid knowledge was assessed using four *ad hoc* statements with three response options (True/False/Do not know), resulting in a score ranging from 0 (no correct answer) to 4 (four correct answers). The statements were the following ones: (1) “Cannabidiol (CBD) is an active ingredient naturally present in the cannabis plant”; (2) “Cannabidiol (CBD) can impair some mental abilities (induce a high)”; (3) “The active ingredient tetrahydrocannabinol (THC) can impair some mental abilities (induce a high)”; (4) “Cannabidiol (CBD) is illegal in France” (Supplementary Table 1).

In the survey, cannabis was defined as containing THC above the French authorized threshold (0.3%), while CBD was defined as any product containing CBD with THC levels at or below this threshold.

We considered acceptability as a construct that reflects the extent to which providers deem cannabis-based products to be appropriate in the context of PD.^45^ Acceptability levels of cannabis and CBD were assessed using two questions for each: (1) “Might you encourage the use of (quality-controlled) medical cannabis [respectively, CBD] for Parkinson’s disease if it were only available on prescription?”; and (2) “Might you encourage the use of (quality-controlled) medical cannabis [respectively, CBD] for Parkinson’s disease if it were available without prescription (ie, over the counter)?” Our assessment of acceptability was therefore expected to encompass to some extent the component constructs of “affective attitude,” “ethicality,” and “perceived effectiveness.”^45^ Respondents could answer Yes/No/Do not know to each question. For each of the two substances, acceptability was categorized as “moderate” for one “Yes” answer, “high,” when both answers were “Yes,” and “low” for all other situations.

The perceived risk of cannabis dependence was assessed by asking: “In your opinion, how great is the risk of becoming dependent on cannabis?” with six possible response options. Participants’ views on the current legal status of cannabis in France were evaluated through two questions: “Are you in favor of easing legal restrictions on the medical use of cannabis in France?” and “Are you in favor of easing legal restrictions on the nonmedical use of cannabis in France?,” each with three possible responses. Barriers to the therapeutic use of cannabis and CBD for PD were assessed using the question: “What are the barriers currently preventing you from agreeing with the use of cannabis [respectively CBD] for therapeutic purposes for Parkinson’s disease (that is to say, outside of official recommendations)?” Participants were asked to select and rank between one and five options from a list of fifteen choices for cannabis and thirteen choices for CBD, respectively.

Participants were required to answer the questions in the order they appeared in order to proceed through the questionnaire. As a result, there were no missing data. The questionnaire was revised and approved by a neurologist. Questionnaire items are provided in Supplementary Table 2.

### Statistical analyses

Participants’ characteristics were compared based on their occupation which we dichotomized into two groups: physicians and nonphysicians. This choice was based on the assumption that physicians would have different views on cannabis-based products as they are the only professionals who can prescribe medications. They are also expected to be aware of the state of the art regarding the efficacy of cannabis-based products, as well as of possible drug-drug interactions. Chi-square and Mann-Whitney (or Student’s) tests were used for categorical and continuous variables, respectively, with *P*-values adjusted for multiple comparisons using the Bonferroni correction.

Two separate binary logistic regression models were run—one for cannabis and the other for CBD—using a binary outcome: moderate/high versus low acceptability (with low acceptability as the reference category). Dichotomization was chosen to mitigate the risk of unstable estimates and poor model convergence, common challenges in regression when sample sizes are small. This approach ensured adequate statistical power and reliable coefficient estimation while maintaining the interpretability of the results. The main theoretically grounded explanatory variables were occupation (ie, physician or nonphysician), cannabinoid knowledge (scored from 0 to 4), and perception of the risk of cannabis dependence. Other explanatory variables included age, gender, city size, practice sector (ie, public or private), number of PD patients, and years of experience managing PD patients. The internal consistency of the cannabinoid knowledge variable was assessed using Cronbach’s alpha, and dichotomization was considered based on the variable’s sample distribution to ensure an adequate events-per-variable (EPV) ratio and avoid overly sparse categories. Similarly, merging modalities of the risk perception variable was considered for categories representing less than 10% of participants. No variables were centered or standardized. Multicollinearity was assessed using variance inflation factors (VIFs) for all candidate explanatory variables.

Variables were initially selected based on a *P*-value threshold of <0.20 (Wald test) in univariable analyses. A backward selection procedure was then employed to finalize the two initial multivariable models, with a significance threshold of 0.05. To ensure comparability, a common set of variables was included in the final multivariable models. Any variable retained in at least one of the initial multivariable models was retained in the two final models, regardless of its *P*-value. The goodness-of-fit of the final models was assessed using the Hosmer–Lemeshow test. As sensitivity analyses, we similarly performed two ordinal logistic regressions using the three-level outcome (low/moderate/high acceptability). As a second sensitivity analysis, we forced the three main explanatory variables (occupation, knowledge, and risk perception) into the final binary regression model, regardless of their *P*-values. Answers to the two questions on position regarding the current legal status of cannabis in France and barriers to its use were compared between the two occupational groups using a Chi-square test.

Analyses were not weighted for representativeness as there is no publicly available national database reporting the characteristics of the French healthcare providers as a whole. Stata/SE 16.1 software (StataCorp LP) was used for all analyses.

## Results

### Study sample characteristics

Three hundred and twenty-four people provided their consent at the beginning of the survey. The survey was completed by 218 participants (79.4% women, median [interquartile range (IQR)] age 41 [34;50] years (Table 1). While the France Parkinson mailing list included approximately 5,000 recipients, we lacked data on the size of the other mailing lists, the proportion of eligible individuals within them, and the audience reach of the websites used for dissemination. Consequently, we cannot accurately estimate the response rate.

**Table 1:** Study sample characteristics according to their occupation (physicians/nonphysicians) (n = 218).

Variables	Total sample N (column %)	Physicians N (column %)	Nonphysicians N (column %)	*P*-value^1^
Total	218 (100)	45 (100)	173 (100)	
Gender				<0.001^2^
Men	45 (20.6)	22 (48.9)	23 (13.3)	
Women	173 (79.4)	23 (51.1)	150 (86.7)	
Age (in years, median [IQR])	41 [34;50]	47 [39;53]	40 [32;50]	0.033^3^
Age (in years, by quartile)				0.649^2^
23–34	58 (26.6)	6 (13.3)	52 (30.1)	
35–41	54 (24.8)	11 (24.4)	43 (24.9)	
42–50	52 (23.9)	11 (24.4)	41 (23.7)	
51–75	54 (24.8)	17 (37.8)	37 (21.4)	
Area of residence				<0.001^2^
Rural area	63 (28.9)	1 (2.2)	62 (35.8)	
Medium-sized city	87 (39.9)	16 (35.6)	71 (41.0)	
Large city (>200 000 inhabitants)	68 (31.2)	28 (62.2)	40 (23.1)	
Type of practice				<0.001^2^
Public only	60 (27.5)	38 (84.4)	22 (12.7)	
Private (with or without public practice)	158 (72.5)	7 (15.6)	151 (87.3)	
Number of PD patients currently followed				<0.001^2^
<10	152 (69.7)	3 (6.7)	149 (86.1)	
≥10	66 (30.3)	42 (93.3)	24 (13.9)	
Number of years of experience with people with PD				0.353^2^
<5	44 (20.2)	5 (11.1)	39 (22.5)	
5–9	45 (20.6)	6 (13.3)	39 (22.5)	
10–19	71 (32.6)	15 (33.3)	56 (32.4)	
≥20	58 (26.6)	19 (42.2)	39 (22.5)	
Cannabinoid knowledge (median [IQR])	4 [3;4]	4 [3;4]	4 [3;4]	0.561^3^
Cannabinoid knowledge (mean)	3.25	3.55	3.17	0.401^4^
Cannabinoid knowledge score (0–4)				1.000^2^
<4	94 (43.1)	15 (33.3)	79 (45.7)	
4	124 (56.9)	30 (66.7)	94 (54.3)	
In your opinion, how great is the risk of becoming dependent on cannabis?				1.000^2^
There is no risk	5 (2.3)	0 (0)	5 (2.9)	
Weak	21 (9.6)	7 (15.6)	14 (8.1)	
Moderate	51 (23.4)	14 (31.1)	37 (21.4)	
Serious	81 (37.2)	18 (40.0)	63 (36.4)	
Very serious	45 (20.6)	5 (11.1)	40 (23.1)	
I do not know	15 (6.9)	1 (2.2)	14 (8.1)	
Cannabis acceptability status				<0.001^2^
Low acceptability	60 (27.5)	26 (57.8)	34 (19.7)	
Moderate acceptability	108 (49.5)	16 (35.6)	92 (53.2)	
High acceptability	50 (22.9)	3 (6.7)	47 (27.2)	
Cannabidiol acceptability status				<0.001^2^
Low acceptability	26 (11.9)	15 (33.3)	11 (6.4)	
Moderate acceptability	80 (36.7)	19 (42.2)	61 (35.3)	
High acceptability	112 (51.4)	11 (24.4)	101 (58.4)	
Are you in favor of alleviating legal restrictions on medical use of cannabis in France?				<0.001^2^
No	12 (5.5)	8 (17.8)	4 (2.3)	
Yes, under certain conditions	136 (62.4)	32 (71.1)	104 (60.1)	
Yes, absolutely	63 (28.9)	5 (11.1)	58 (33.5)	
No opinion	7 (3.2)	0 (0)	7 (4.1)	
Are you in favor of alleviating legal restrictions on nonmedical use of cannabis in France?				1.000^2^
No	104 (47.7)	23 (51.1)	81 (46.8)	
Yes	56 (25.7)	14 (31.1)	42 (24.3)	
No opinion	58 (26.6)	8 (17.8)	50 (28.9)	

IQR, interquartile range; PD, Parkinson’s disease.

1 *P*-values were adjusted for multiple comparisons using the Bonferroni correction.

2 Chi-square test.

3 Mann-Whitney test.

4 Student’s t-test.

Participants accessed the survey through various channels, listed here in descending order of frequency: “other” channels (39.0%), the France Parkinson association (26.6%), neurology networks (20.6%), and healthcare providers (13.8%). No further details were collected for the “other” category, as the survey did not prompt respondents to specify their recruitment source. The study population comprised 82 speech therapists, 58 physiotherapists, 45 physicians (including 42 neurologists), 21 nurses, three dieticians, three occupational therapists, two neuropsychologists, one pharmacist, one clinical psychologist, one psychomotor therapist, and one naturopath. Physicians therefore represented 20.6% of the study population; they did not differ significantly from other participants in terms of perceived risk of becoming dependent on cannabis, nor in their cannabinoid knowledge (Table 1).

One quarter (27.5%) of the study population had low acceptability of cannabis for PD management; 11.9% had low acceptability for CBD (Table 1, Figure). The proportion of physicians with low acceptability was higher compared to nonphysicians for both substances (*P* < 0.001, z-proportion test). The proportion of low acceptability was significantly higher for cannabis than for CBD in both sub-populations (*P* ≤ 0.020, z-proportion test). Answers to the separate acceptability questions can be found in Supplementary Table 3. Cronbach’s alpha of the cannabinoid knowledge variable was of 0.656. As 56.9% of the sample had a score of 4, the score was dichotomized (<4 vs 4).

**Figure:**
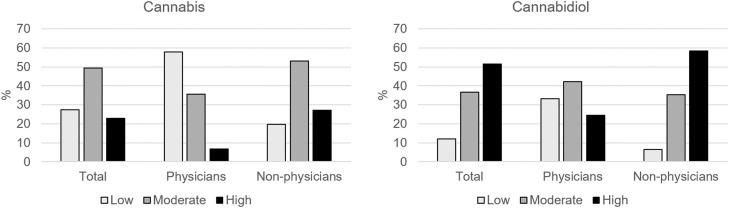
Proportions of cannabis and cannabidiol acceptability status according to participants’ occupation (physicians, n = 45; nonphysicians, n = 173).

### Factors associated with acceptability of medical cannabis and CBD

The results of the multivariable analyses are summarized in Table 2. All VIF values ranged from 1.15 to 3.35, suggesting no collinearity issue. The EPV ratio was 12, which exceeds the commonly recommended threshold of 10 to minimize the risks of overfitting, bias, and instability in parameter estimates.^46^ For both cannabis and CBD, moderate/high acceptability was inversely associated with being a physician (adjusted odds ratio (aOR) [95% confidence interval (CI)] 0.13 [0.06;0.28], *P* < 0.001; 0.10 [0.04;0.27], *P* < 0.001, respectively). Participants who considered that there was a very serious risk of becoming dependent on cannabis were 75% less likely to report moderate/high cannabis acceptability (aOR [95% CI] 0.26 [0.08;0.88], *P* = 0.031).

**Table 2:** Factors associated with high or moderate cannabis and cannabidiol acceptability levels (n = 218, multivariable logistic regression models).

	Cannabis		Cannabidiol	
	aOR [95% CI]	*P*-value	aOR [95% CI]	*P*-value
Healthcare occupation				
Nonphysician (ref.)	1		1	
Physician	0.13 [0.06;0.28]	<0.001	0.10 [0.04;0.27]	<0.001
In your opinion, how great is the risk of becoming dependent on cannabis?				
There is no risk/Small risk (ref.)	1		1	
Moderate risk	1.30 [0.37;4.59]	0.681	1.46 [0.33;6.46]	0.615
Serious risk	0.67 [0.21;2.12]	0.500	1.56 [0.38;6.35]	0.535
Very serious risk	0.26 [0.08;0.88]	0.031	1.18 [0.23;5.91]	0.843
I do not know	0.45 [0.09;2.22]	0.330	0.23 [0.04;1.30]	0.096

aOR, adjusted odds ratio; CI, confidence interval.

Hosmer–Lemeshow chi-square = 1.50 (*P* = 0.913) for cannabis; Hosmer–Lemeshow chi-square = 0.46 (*P* = 0.977) for cannabidiol.

Ordinal logistic regression yielded consistent results. For both substances, higher acceptability level was inversely associated with being a physician. Participants who considered that there was a serious or very serious risk of becoming dependent on cannabis were less likely to report higher cannabis acceptability level. Furthermore, a good cannabinoid knowledge was positively associated with CBD acceptability (1.98 [1.13;3.48], *P* = 0.017), but not with cannabis acceptability (*P* = 0.126) (Supplementary Table 4). Sensitivity analysis forcing the cannabinoid knowledge variable into the final model yielded results similar to the ones found in main analyses (acceptability inversely associated with being a physician for both substances; perceiving a very serious risk of dependence inversely associated with cannabis acceptability). Knowledge was not associated with the outcomes (*P* > 0.3, data not shown).

Given the relatively low number of physicians in our sample, we conducted, as *post hoc* sensitivity analysis, a Firth penalized logistic regression on the binary outcomes. Results were very similar to the one obtained in main analyses (Supplementary Table 5).

### Positions regarding the legal status of cannabis and barriers to use

Descriptive analyses showed that the vast majority (91.3%) of participants supported easing legal restrictions on medical cannabis use in France, whether absolutely or under certain conditions (Table 1). However, support for reducing restrictions on nonmedical use was considerably lower, at 25.7%. Inferential analyses showed that physicians were significantly less likely to be “absolutely” in favor of easing legal restrictions on medical cannabis use (11.1% vs 33.5% in the rest of the sample, *P* = 0.038).

Descriptively, the top three barriers to agreeing with the use of therapeutic cannabis for PD as reported by physicians were: “the absence of recommendations from medical authorities” (82.2%), “a lack of evidence to support its effectiveness” (80.0%), and “a fear of psychoactive effects (ie, drug *highs*)” (62.2%). Among nonphysicians, the most cited barriers were “a fear of drug-drug interactions” (63.0%), “a lack of information” (61.8%), and “the absence of recommendations from medical authorities” (50.3%) (Table 3).

**Table 3:** Barriers to therapeutic use of cannabis and cannabidiol of participants according to their occupation (ie, physicians vs nonphysicians) (n = 218).

	Cannabis			Cannabidiol		
	Physicians	Nonphysicians	Chi-square test *P*-value	Physicians	Nonphysicians	Chi-square test *P*-value
	Cited N (%)	Cited N (%)		Cited N (%)	Cited N (%)	
Putting oneself or one’s patients in an illegal situation	14 (31.1)	86 (49.7)	0.026	-	-	-
Fear that patients would become dependent on the substance	16 (35.6)	62 (35.8)	0.972	2 (4.4)	26 (15.0)	0.059
Fear of psychoactive effects (ie, drug *highs*)	28 (62.2)	85 (49.1)	0.117	5 (11.1)	20 (11.6)	0.933
Fear of drug-drug interactions	6 (13.3)	109 (63.0)	0.000	7 (15.6)	103 (59.5)	0.000
Fear of other adverse effects	20 (44.4)	81 (46.8)	0.776	13 (28.9)	89 (51.4)	0.007
Lack of evidence to support its effectiveness	36 (80.0)	36 (20.8)	0.000	35 (77.8)	73 (42.2)	0.000
Lack of information about proper usage	16 (35.6)	107 (61.8)	0.002	24 (53.3)	135 (78.0)	0.001
Difficulties in supply	4 (8.9)	20 (11.6)	0.610	2 (4.4)	11 (6.4)	0.629
Cost of substance	4 (8.9)	31 (17.9)	0.142	12 (26.7)	47 (27.2)	0.946
The lack of recommendations from medical authorities	37 (82.2)	87 (50.3)	0.000	38 (84.4)	106 (61.3)	0.003
My colleagues’ reluctance	0(0)	0(0)	-	1 (2.2)	0 (0)	-
My relatives' reluctance (other than colleagues)	0(0)	0(0)	-	0 (0)	2 (1.2)	-
Fear of stigmatization (social disapproval) of patients	1 (2.2)	9 (5.2)	-	0 (0)	13 (7.5)	-
Fear of stigmatization (social disapproval) of myself	0 (0)	1 (0.6)	-	0 (0)	5 (2.9)	-
Its form/mode of administration (eg, dried herb/resin) is poorly adapted to certain patients	2 (4.4)	28 (16.2)	0.042	-	-	-

Percentages indicate the proportion of the column population who cited the barrier.

Similarly, descriptive results indicated that the three most cited barriers to agreeing with the use of therapeutic CBD for PD were “the absence of recommendations from medical authorities” (84.4%), “a lack of evidence to support its effectiveness” (77.8%), and “a lack of information about proper usage” (53.3%) for physicians. Among nonphysicians, the most cited barriers were “a lack of information about proper usage” (78.0%), “the absence of recommendations from medical authorities” (61.3%), and “a fear of drug-drug interactions” (59.5%) (Table 3).

Further descriptive analyses showed that, among physicians, “a lack of evidence to support its effectiveness” was overwhelmingly the primary barrier (ie, cited on first position) to agreeing with the use of both cannabis and CBD for the therapeutic management of PD, while no singular primary barrier stood out among nonphysicians (Supplementary Table 6).

## Discussion

This study is the first to explore French healthcare providers’ acceptability, knowledge and perceptions of therapeutic cannabis and CBD use for PD. Among participants, physicians—who are the only professionals authorized to prescribe medications—exhibited lower acceptability of cannabis use compared to other healthcare providers, with a lack of evidence to support its effectiveness emerging as a major barrier. A similar disparity between physicians and nonphysicians was observed regarding the acceptability of CBD use. Notably, CBD was more widely accepted than cannabis across both groups.

### Comparison with prior studies

Previous research has highlighted differences in attitudes toward medical cannabis between physicians and nonphysicians. In their systematic review of healthcare providers’ beliefs, knowledge, and concerns about medical cannabis, Gardiner et al reported that nurses and other allied health professionals were “largely supportive of medicinal cannabis.” No such result was given for medical practitioners.^29^ Similarly, a study involving clinicians in the District of Columbia revealed that nurse practitioners were more likely than oncologists to recognize a medical role for cannabis.^30^ Another systematic review indicated that physicians with specialties—reflecting our interviewed sample of physicians—were less supportive of the legal use of medical cannabis.^27^ Our findings extend these observations by including attitudes toward CBD-based products.

Few studies have specifically explored healthcare providers’ attitudes toward the therapeutic use of cannabis for PD. In our study, acceptability was assessed using hypothetical scenarios (eg, “Might you encourage […] if […]”), which were tailored to the French context. Since regulatory and cultural contexts vary across countries, so too do the formulations used to evaluate acceptability among healthcare providers. Consequently, the acceptability levels we observed are challenging to compare directly with those reported in other studies, where access to cannabis-based products may differ substantially. For example, Bega et al reported that among an international sample of 56 PD neurologists, 10% had recommended cannabis use to their patients, with 39% endorsing its use when requested by patients.^28^ In a quota-based survey conducted among US-based neurologists, pharmacists, nurses, and nurse practitioners, Szaflarski et al revealed that neurologists held less favorable views toward therapeutic cannabis for PD compared to nurses and nurse practitioners. They also observed that attitudes across all surveyed professions were linked to their knowledge of cannabis.^31^

Healthcare providers’ opinions on the legalization of medical cannabis can play a pivotal role in shaping political decisions regarding its authorization. In our study, we identified marked differences in attitudes between physicians and nonphysicians. Specifically, while 82.2% of physicians supported easing legal restrictions on medical cannabis use in France, 93.6% of other healthcare providers endorsed this stance. Szaflarski et al reported that over 80% of healthcare providers in their study agreed that cannabis products for medical purposes should be legal if prescribed by a medical provider.^31^ Similarly, in Bega et al’s study, nearly 70% of neurologists expressed belief that cannabis should be permissible for medicinal prescribing.^28^

### Potential explanations

In our convenience sample, the less favorable attitude of physicians towards the therapeutic use of cannabis-based products for PD may stem partially from their deeper understanding of potential adverse effects, such as cognitive impairment^47^ and drug-drug interactions,^23^ which could be prevalent among self-medicated patients (ie, whether for PD or another condition).^48^ This may thus contribute to a heightened risk perception among physicians, extending beyond concerns about dependence. However, the primary barriers to agreeing with the use of cannabis and CBD for the therapeutic management of PD cited by physicians in our study seemed to be largely driven by the lack of robust scientific evidence and official recommendations. These barriers, less frequently reported by nonphysicians, have been reported as barriers to prescribing medical cannabis for chronic pain in previous research.^32^ Indeed, given that under French law, physicians are free to prescribe treatments based on the best available scientific evidence, encouraging the use of cannabis-based products in the absence of scientific proof may raise issues of professional liability.

Despite we checked for multicollinearity, we cannot definitely discard potential confounding effects to explain lower acceptability levels among physicians. The fact that in our sample physicians were older may have had an effect on their acceptability, as older age may be correlated with higher risk perception in terms of cannabis use.^49^, ^50^, ^51^ Physicians were also more likely to be male, a characteristic that was positively associated with attitudes regarding cannabis-base therapies in Szaflarski et al’s work,^31^ but not associated with believing that cannabis has medicinal use nor recommending cannabis to patients in Schauer et al’s work.^30^ In a previous work not focused on PD, female providers appeared more likely to be supportive of medical cannabis than male providers.^52^ In our sample, physicians were also less likely to live in a rural area.

A previous study involving French general practitioners found that having a good knowledge of the effects of cannabinoids was associated with a willingness to become a medical cannabis prescriber.^53^ Consequently, the low acceptability of cannabis-based products among physicians in our study may also be partly attributable to knowledge gaps. This is supported by the significant number of respondents who identified insufficient information on proper usage as a barrier. Similarly, concerns about drug-drug interactions, though less frequently cited, may also reflect a lack of familiarity with these risks. These findings align with those of Rønne et al, who also highlighted a knowledge gap among physicians in their study.^27^ In sensitivity analyses, we found a positive effect of cannabinoid knowledge on acceptability of CBD (regardless of the occupation). As we did not find such an effect for cannabis, we may make the hypothesis that acceptability of CBD is related to the awareness of its relatively safe profile.

Regardless of provider type (ie, physicians or nonphysicians), we found that perceiving cannabis dependence as a very serious risk was consistently associated with lower acceptability of medical cannabis for PD. This finding aligns with a previous study among people living with PD in France,^44^ as well as with Bega et al’s research, where 84% of participants perceived cannabis as potentially addictive.^28^ A recent meta-analysis by Dawson et al estimated the prevalence of cannabis use disorder at 25% among users of medical cannabis.^54^ However, it included participants using cannabis for “medicinal reasons,” a term which does not necessarily reflect medical supervision (ie, vs self-medication) of dosages and frequencies. Moreover, their meta-analysis did not exclude dual use motives (ie, recreational and medical). The authors also noted that younger age may be a risk factor for cannabis use dependence, which is important when considering that PD affects older people. Importantly, appropriate clinician training could mitigate dependence risks by implementing key recommendations such as proper screening of patients and monitoring of medical cannabis use.^55^

The hesitance among our convenience sample of French physicians to support loosening legal restrictions on medical cannabis may be rooted in skepticism regarding its therapeutic benefits, as highlighted by our analysis of barriers to its use. A study of general practitioners in France also suggested that physicians might oppose the legalization of medical cannabis due to concerns over potential risks to their own security.^34^ Furthermore, physicians expressed apprehension that legalizing medical cannabis could pave the way for broader legalization of nonmedical cannabis use.^34^ Moreover, the ongoing national experiment—which focuses on the feasibility of prescribing cannabis-based products rather than their effectiveness—may have heightened physicians' reluctance. In this context, cannabis is treated as a last-resort option for a limited set of conditions, and the program’s future remains uncertain. Patients enrolled in this program can be monitored until March 2026, while the regulatory framework for medical cannabis production and authorization was submitted to the European Commission in March 2025. Additionally, the complexity and heterogeneity of EU regulations on medical cannabis^56^ may discourage healthcare providers from considering cannabis-based products as reliable therapeutic options.

### Implications of the findings

Currently, French healthcare curricula do not include formal education on cannabis-based products. Yet, previous studies have demonstrated that brief educational interventions can significantly improve healthcare providers’ knowledge and positively influence their attitudes toward cannabis-based medicines.^48^^,^^57^ Accordingly, integrating such training into healthcare education programs could address the need for a deeper understanding of cannabis-based products^28^^,^^29^ while also helping to correct misconceptions,^30^^,^^34^ with the broader goal of enhancing patient QoL.

In our sample, we found that nonphysician healthcare providers generally held a positive view towards the use of cannabis-based products; whereas physicians were less supportive. Should the French regulatory framework evolve to permit therapeutic access to cannabis-based products, our results suggest that a model granting physicians discretionary authority to determine specific prescription indications—though common in many jurisdictions^58^—may prove insufficient to ensure equitable access for people with PD. Instead, our data suggest a need for evidence-based guidelines issued by medical institutions, coupled with targeted training programs, to secure physician endorsement of such practices. This requirement appears particularly crucial for cannabis compared to CBD, suggesting a more nuanced situation for CBD.

As long as CBD and cannabis remain somehow accessible outside of medical prescriptions in France, physicians—regardless of their personal views—must proactively engage in open discussions about these substances with people living with PD. This proactive approach is necessary to prevent or mitigate potential adverse effects associated with the use of cannabis-based products. Without a nonjudgmental and supportive exchange, patients may choose to hide their use from physicians,^24^^,^^59^^,^^60^ or turn to nonphysician healthcare providers, who tend to be more receptive to cannabis-based therapies. While patient-reported data would be needed to confirmed it, this latter assumption is somehow supported by our provider-reported results. For instance, nurses generally hold more positive attitudes toward cannabis-based products, potentially reducing stigma compared to physicians.^33^^,^^61^ However, these professionals may lack in-depth training on cannabis-based products, limiting their ability to provide comprehensive patient counseling—particularly regarding drug interaction risks. Given the relatively higher acceptability of CBD compared to cannabis, we hypothesize that healthcare professionals may perceive CBD as a more accessible entry point for initiating discussions with patients about cannabis-based products.

Balancing evidence-based practice with openness to evolving therapies is therefore a key ethical challenge for healthcare providers, particularly in PD where unmet needs persist. While the current lack of consensual and robust evidence for cannabis and CBD necessitates caution, dismissing patient interest outright may risk undermining trust and overlooking potential future benefits. Encouraging transparent discussions about the limits of existing evidence—while remaining receptive to new data—can foster a patient-centered approach that respects both scientific rigor and individual preferences.

### Strengths and limitations

The primary strength of our study lies in its pioneering nature, being the first to investigate healthcare providers' perspectives on cannabis-based products for PD within France’s restrictive legal framework. Additionally, we successfully collected and compared responses from both physicians and nonphysician healthcare providers, thereby revealing distinctions based on occupation. Furthermore, our study comprehensively assessed both cannabis and CBD, with the latter receiving comparatively less scrutiny regarding its acceptability among healthcare providers.

However, the relatively modest sample size—particularly within the physician subgroup—may have limited our ability to identify other factors influencing the acceptability of cannabis and CBD. It may also have contributed to the wide confidence intervals observed for some estimates, warranting cautious interpretation of these results. The cross-sectional nature of the study prevents from any causal inference. Moreover, the broad range of professions within the ‘nonphysician’ category necessitates a careful interpretation and cautious generalization of our findings, as does the disproportionate sample size between physicians and nonphysicians. The professional heterogeneity within the nonphysician group may have obscured occupation-specific differences, given that healthcare providers—depending on their professional roles—may prioritize symptoms with varying expected responsiveness to cannabis-based products. Additionally, our method did not allow for assessing the response rate or potential recruitment bias, as we relied on word-of-mouth dissemination and did not target a finite group of individuals. Online recruitment may indeed introduce a selection bias toward professionals who are involved in collective organizations and already interested in the cannabis topic. While the online and anonymous nature of the survey likely mitigated response bias, we cannot rule out the possibility of socially desirable answering, especially in this professional context and given the sensitivity of this topic in France. Results from our convenience sample cannot be generalized to French healthcare providers and thus call for further research to validate and expand upon our findings. Finally, as we did not use validated tools, the psychometric properties of our questionnaire remain to be established, especially regarding the acceptability component constructs that were captured by the questions we used (based on hypothetical scenarios rather than actual clinical behavior), or the multidimensional nature of cannabinoid knowledge. Since this latter variable was not validated, the associated findings should be interpreted as exploratory.

Regarding our acceptability variable, as it relied on two scenarios (prescription vs over-the-counter), it may have partially captured regulatory attitudes rather than strict therapeutic acceptability. For analytical and interpretability purposes, we collapsed answers to the two outcome questions into three acceptability levels, and then dichotomized the variable. Such data transformation may have obscured nuance, as several combinations of answers could yield to moderate and low acceptability levels. However, as the low acceptability category included participants who did not provide any positive answer to the two questions, we expect such a dichotomization to be relatively pertinent.

## Conclusion

Our exploratory study highlights that among a sample of healthcare providers in France, physicians showed lower acceptability towards cannabis and CBD for therapeutic use in PD compared to other healthcare professions. CBD was generally more accepted than cannabis. If cannabis-based products were to be authorized for symptom alleviation in PD patients, physicians may require more robust scientific evidence and training before prescribing these treatments.

## Funding

This work was supported by the association France Parkinson.

## Declaration of competing interest

The authors declare the following financial interests/personal relationships which may be considered as potential competing interests: Tangui Barre reports financial support was provided by Association France Parkinson. Other authors declare that they have no known competing financial interests or personal relationships that could have appeared to influence the work reported in this paper.

## Appendix Supplementary materials

Image, application 1

Image, application 2

Image, application 3

Image, application 4

Image, application 5

Image, application 6
