# Supplementary material for: Healthcare Providers’ Acceptability of Cannabis And Cannabidiol to Manage Parkinson’s Disease in France
Source: Curr Ther Res Clin Exp. 2026 Apr 3;104:100830. doi: 10.1016/j.curtheres.2026.100830 (PMC13141070; doi:10.1016/j.curtheres.2026.100830)
Supplement: Supplementary file 1 [file mmc1.docx]

**Supplementary Table 1. Answers to *ad hoc* statements assessing knowledge about cannabinoids, according to participants’ occupation (i.e., physicians vs. non-physicians) (n=218)**

|  | **Total sample**  **N (%)** | **Physicians**  **N (%)** | **Non-physicians**  **N (%)** | **p-value^1^** |
| --- | --- | --- | --- | --- |
| **Cannabidiol (CBD) is an active ingredient naturally present in the cannabis plant** |  |  |  | 0.122 |
| *True* | 195 (89.4) | 44 (97.8) | 151 (87.3) |  |
| False | 3 (1.4) | 0 (0) | 3 (1.7) |  |
| I do not know | 20 (9.2) | 1 (2.2) | 19 (11) |  |
| **Cannabidiol (CBD) can impair some mental abilities (induce a *high*)** |  |  |  | 0.098 |
| True | 7 (3.2) | 3 (6.7) | 4 (2.3) |  |
| *False* | 167 (76.6) | 37 (82.2) | 130 (75.1) |  |
| I do not know | 44 (20.2) | 5 (11.1) | 39 (22.5) |  |
| **The active ingredient tetrahydrocannabinol (THC) can impair some mental abilities (induce a *high*)** |  |  |  | 0.033 |
| *True* | 156 (71.6) | 39 (86.7) | 117 (67.6) |  |
| False | 12 (5.5) | 2 (4.4) | 10 (5.8) |  |
| I do not know | 50 (22.9) | 4 (8.9) | 46 (26.6) |  |
| **Cannabidiol (CBD) is illegal in France** |  |  |  | 0.948 |
| True | 10 (4.6) | 2 (4.4) | 8 (4.6) |  |
| *False* | 191 (87.6) | 40 (88.9) | 151 (87.3) |  |
| I do not know | 17 (7.8) | 3 (6.7) | 14 (8.1) |  |

Correct answers are italicized.

**^1^** Chi-square test
