# Supplementary material for: Healthcare Providers’ Acceptability of Cannabis And Cannabidiol to Manage Parkinson’s Disease in France
Source: Curr Ther Res Clin Exp. 2026 Apr 3;104:100830. doi: 10.1016/j.curtheres.2026.100830 (PMC13141070; doi:10.1016/j.curtheres.2026.100830)
Supplement: Supplementary file 2 [file mmc2.docx]

**Supplementary Table 2. Questionnaire items and possible answers**

| **Question** | **Possible answers** |
| --- | --- |
| How old are you ? | _____ (numerical answer in years) |
| You are : | - A woman - A man - Other |
| You live : | - **In a large city, i.e., with more than 200,000 inhabitants (such as Paris, Marseille, Lyon, Toulouse, Nice, Nantes, Montpellier, Strasbourg, Bordeaux, Lille, Rennes) or its suburbs** - In a medium-sized city - In a rural area |
| **Your department number of residence** | _____ (numerical answer) |
| You are : | - Neurologist - General practitioner - Physician, other specialty: _____ - Physiotherapist - Speech therapist - Nurse - Other healthcare professional category: _____ |
| **Where do you practice?** | - In private practice - In a public institution - Both |
| **How many people with Parkinson's disease are you currently following?** | - Fewer than 10 - Between 10 and 15 (inclusive) - Between 16 and 20 (inclusive) - More than 20 |
| **How long have you been managing for people with Parkinson’s disease?** | _____ (numerical answer in years) |
| Cannabidiol (CBD) is an active ingredient naturally present in the cannabis plant^1^ | - True - False - I do not know |
| Cannabidiol (CBD) can impair some mental abilities (induce a *high*)^1^ | - True - False - I do not know |
| The active ingredient tetrahydrocannabinol (THC) can impair some mental abilities (induce a *high*)^1^ | - True - False - I do not know |
| Cannabidiol (CBD) is illegal in France^1^ | - True - False - I do not know |
| **Cannabis is a plant that naturally contains THC and cannabidiol. THC is a compound that alters mental capacities, whereas cannabidiol is a compound that does not have this effect. For this reason, products containing cannabidiol but very little THC (less than 0.3%) are not prohibited, while traditional cannabis, which contains THC (more than 0.3%), is illegal. However, there is cannabis that is high in cannabidiol and very low in THC.**  **In the rest of this questionnaire, the term ‘cannabis’ refers to cannabis containing THC, as well as its derived products that also contain THC above the authorized thresholds.**  **In the rest of this questionnaire, cannabidiol will be abbreviated as CBD, and this term will refer to products (regardless of their form) containing cannabidiol but not containing THC above the authorized thresholds.** | |
| Might you encourage the use of (quality-controlled) medical cannabis for Parkinson’s disease if it were only available on prescription?^2^ | - **Yes** - **No** - **Do not know** |
| Might you encourage the use of (quality-controlled) medical CBD for Parkinson’s disease if it were only available on prescription?^2^ | - **Yes** - **No** - **Do not know** |
| Might you encourage the use of (quality-controlled) medical cannabis for Parkinson’s disease if it were available without prescription (i.e., over the counter)?^2^ | - **Yes** - **No** - **Do not know** |
| Might you encourage the use of (quality-controlled) medical CBD for Parkinson’s disease if it were available without prescription (i.e., over the counter)?^2^ | - **Yes** - **No** - **Do not know** |
| What are the barriers currently preventing you from agreeing with the use of cannabis for therapeutic purposes for Parkinson’s disease (that is to say, outside of official recommendations)?  *Up to 5 responses possible; please rank them from most important to least important (1 being the most important).* | - Putting oneself or one’s patients in an illegal situation - Fear that patients would become dependent on the substance - Fear of psychoactive effects (i.e., drug *highs*) - Fear of drug-drug interactions - Fear of other adverse effects - Lack of evidence to support its effectiveness - Lack of information about proper usage - Difficulties in supply - Cost of substance - The lack of recommendations from medical authorities - My colleagues’ reluctance - My relatives' reluctance (other than colleagues) - Fear of stigmatization (social disapproval) of patients - Fear of stigmatization (social disapproval) of myself - Its form/mode of administration (e.g., dried herb/resin) is poorly adapted to certain patients |
| What are the barriers currently preventing you from agreeing with the use of CBD for therapeutic purposes for Parkinson’s disease (that is to say, outside of official recommendations)?  *Up to 5 responses possible; please rank them from most important to least important (1 being the most important).* | - Fear that patients would become dependent on the substance - Fear of psychoactive effects (i.e., drug highs) - Fear of drug-drug interactions - Fear of other adverse effects - Lack of evidence to support its effectiveness - Lack of information about proper usage - Difficulties in supply - Cost of substance - The lack of recommendations from medical authorities - My colleagues’ reluctance - My relatives' reluctance (other than colleagues) - Fear of stigmatization (social disapproval) of patients - Fear of stigmatization (social disapproval) of myself |
| In your opinion, how great is the risk of becoming dependent on cannabis? | - There is no risk - Weak - Moderate - Serious - Very serious - I do not know |
| Are you in favor of easing legal restrictions on the medical use of cannabis in France? | - No - Yes - I do not know |
| Are you in favor of easing legal restrictions on the non-medical use of cannabis in France? | - No - Yes - I do not know |

^1^For each of those four questions, only one answer was correct and coded as 1. Other answers were coded as 0 (see **Supplementary Table 1**). Cannabinoid knowledge was computed as the sum of those four questions, resulting in a score ranging from 0 (no correct answer) to 4 (four correct answers). This score was then dichotomized (<4 vs. 4).

^2^For each of those four questions, ‘Yes’ was coded as 1, and ‘No’ and ‘Do not know’ as 0. For cannabis and CBD separately, the two answers were summed, resulting in an acceptability score of 0 (‘low’), 1 (‘moderate’) or 2 (‘high’).
