# Supplementary material for: Healthcare Providers’ Acceptability of Cannabis And Cannabidiol to Manage Parkinson’s Disease in France
Source: Curr Ther Res Clin Exp. 2026 Apr 3;104:100830. doi: 10.1016/j.curtheres.2026.100830 (PMC13141070; doi:10.1016/j.curtheres.2026.100830)
Supplement: Supplementary file 3 [file mmc3.docx]

**Supplementary Table 3. Answers to the two separate acceptability questions according to participants’ occupation (i.e., physicians vs, non-physicians) (n=218)**

|  | **Total sample**  **N (%)** | **Physicians**  **N (%)** | **Non-physicians**  **N (%)** |
| --- | --- | --- | --- |
| **Might you encourage the use of (quality-controlled) medical cannabis for Parkinson's disease if it were only available on prescription?** |  |  |  |
| Yes | 156 (71.6) | 19 (42.2) | 137 (79.2) |
| No | 26 (11.9) | 15 (33.3) | 11 (6.4) |
| Do not know | 36 (16.5) | 11 (24.4) | 25 (14.5) |
| **Might you encourage the use of (quality-controlled) medical CBD for Parkinson's disease if it were only available on prescription?** |  |  |  |
| Yes | 52 (23.9) | 3 (6.7) | 49 (28.3) |
| No | 91 (41.7) | 31 (68.9) | 60 (34.7) |
| Do not know | 75 (34.4) | 11 (24.4) | 64 (37) |
| **Might you encourage the use of (quality-controlled) medical cannabis for Parkinson's disease if it were available without prescription (i.e., over-the-counter)?** |  |  |  |
| Yes | 186 (85.3) | 28 (62.2) | 158 (91.3) |
| No | 8 (3.7) | 6 (13.3) | 2 (1.2) |
| Do not know | 24 (11) | 11 (24.4) | 13 (7.5) |
| **Might you encourage the use of (quality-controlled) medical CBD for Parkinson's disease if it were available without prescription (i.e., over-the-counter)?** |  |  |  |
| Yes | 118 (54.1) | 13 (28.9) | 105 (60.7) |
| No | 47 (21.6) | 20 (44.4) | 27 (15.6) |
| Do not know | 53 (24.3) | 12 (26.7) | 41 (23.7) |
