# Supplementary material for: Healthcare Providers’ Acceptability of Cannabis And Cannabidiol to Manage Parkinson’s Disease in France
Source: Curr Ther Res Clin Exp. 2026 Apr 3;104:100830. doi: 10.1016/j.curtheres.2026.100830 (PMC13141070; doi:10.1016/j.curtheres.2026.100830)
Supplement: Supplementary file 4 [file mmc4.docx]

**Supplementary Table 4. Factors associated with cannabis and cannabidiol acceptability levels (n=218, multivariable ordinal logistic regression models)**

|  | **Cannabis** |  | **Cannabidiol** |  |
| --- | --- | --- | --- | --- |
|  | aOR [95% CI] | p-value | aOR [95% CI] | p-value |
| **Healthcare occupation** |  |  |  |  |
| Non-physician (ref.) | 1 |  | 1 |  |
| Physician | 0.13 [0.06;0.27] | <0.001 | 0.16 [0.09;0.33] | <0.001 |
| **In your opinion, how great is the risk of becoming dependent on cannabis?** |  |  |  |  |
| There is no risk/Small risk (ref.) | 1 |  | 1 |  |
| Moderate risk | 0.78 [0.31;2.00] | 0.610 | 1.01 [0.38;2.70] | 0.989 |
| Serious risk | 0.37 [0.15;0.93] | 0.033 | 0.69 [0.27;1.76] | 0.442 |
| Very serious risk | 0.22 [0.08;0.62] | 0.004 | 1.43 [0.50;4.11] | 0.506 |
| I do not know | 0.59 [0.16;2.23] | 0.439 | 0.54 [0.14;2.13] | 0.383 |
| **Cannabinoid knowledge score of 4 (vs. <4)^1^** | 1.54 [0.89;2.68] | 0.126 | 1.98 [1.13;3.48] | 0.017 |

aOR, adjusted odds ratio; CI, confidence interval

The proportional odds assumption was verified using Brand test (p=0.281 for cannabis, and p=0.132 for cannabidiol)

^1^The score was derived from the answers to four *ad hoc* questions.
