# Supplementary material for: Healthcare Providers’ Acceptability of Cannabis And Cannabidiol to Manage Parkinson’s Disease in France
Source: Curr Ther Res Clin Exp. 2026 Apr 3;104:100830. doi: 10.1016/j.curtheres.2026.100830 (PMC13141070; doi:10.1016/j.curtheres.2026.100830)
Supplement: Supplementary file 5 [file mmc5.docx]

**Supplementary Table 5. Factors associated with high or moderate cannabis and cannabidiol acceptability levels (n=218, Firth penalized multivariable logistic regression models)**

|  | **Cannabis** |  | **Cannabidiol** |  |
| --- | --- | --- | --- | --- |
|  | aOR [95% CI] | p-value | aOR [95% CI] | p-value |
| **Healthcare occupation** |  |  |  |  |
| Non-physician (ref.) | 1 |  | 1 |  |
| Physician | 0.14 [0.06;0.29] | <0.001 | 0.12 [0.05;0.29] | <0.001 |
| **In your opinion, how great is the risk of becoming dependent on cannabis?** |  |  |  |  |
| There is no risk/Small risk (ref.) | 1 |  | 1 |  |
| Moderate risk | 1.32 [0.39;4.47] | 0.659 | 1.48 [0.36;6.12] | 0.586 |
| Serious risk | 0.71 [0.23;2.15] | 0.543 | 1.61 [0.42;6.15] | 0.489 |
| Very serious risk | 0.28 [0.09;0.92] | 0.036 | 1.19 [0.26;5.49] | 0.825 |
| I do not know | 0.46 [0.10;2.10] | 0.314 | 0.24 [0.05;1.28] | 0.096 |

aOR, adjusted odds ratio; CI, confidence interval
