# Supplementary material for: Healthcare Providers’ Acceptability of Cannabis And Cannabidiol to Manage Parkinson’s Disease in France
Source: Curr Ther Res Clin Exp. 2026 Apr 3;104:100830. doi: 10.1016/j.curtheres.2026.100830 (PMC13141070; doi:10.1016/j.curtheres.2026.100830)
Supplement: Supplementary file 6 [file mmc6.docx]

**Supplementary Table 6. Primary barriers to agreeing to the use of cannabis and cannabidiol for the therapeutic management of PD according to participants’ occupation (i.e., physicians vs. non-physicians) (n=218)**

|  | Cannabis |  | Cannabidiol |  |
| --- | --- | --- | --- | --- |
|  | Physicians | Non-physicians | Physicians | Non-physicians |
|  | Cited as primary barrier  N (%) | Cited as primary barrier  N (%) | Cited as primary barrier  N (%) | Cited as primary barrier  N (%) |
| Putting oneself or one’s patients in an illegal situation | 4 (8.9) | 39 (22.5) | - | - |
| Fear that patients would become dependent on the substance | 1 (2.2) | 12 (6.9) | 1 (2.2) | 8 (4.6) |
| Fear of psychoactive effects (drug *highs*) | 6 (13.3) | 23 (13.3) | 0 (0) | 3 (1.7) |
| Fear of drug-drug interactions | 0 (0) | 27 (15.6) | 0 (0) | 28 (16.2) |
| Fear of other adverse effects | 2 (4.4) | 5 (2.9) | 3 (6.7) | 2 (1.2) |
| Lack of evidence to support its effectiveness | 24 (53.3) | 8 (4.6) | 27 (60) | 29 (16.8) |
| Lack of information about proper usage | 1 (2.2) | 36 (20.8) | 4 (8.9) | 64 (37) |
| Difficulties in supply | 0 (0) | 0 (0) | 0 (0) | 1 (0.6) |
| Cost of substance | 0 (0) | 4 (2.3) | 2 (4.4) | 9 (5.2) |
| The absence of recommendations from medical authorities | 7 (15.6) | 16 (9.2) | 8 (17.8) | 28 (16.2) |
| My colleagues’ reluctance | 0 (0) | 0 (0) | 0 (0) | 0 (0) |
| My relatives' reluctance (other than colleagues) | 0 (0) | 0 (0) | 0 (0) | 0 (0) |
| Fear of stigmatization (social disapproval) of patients | 0 (0) | 0 (0) | 0 (0) | 1 (0.6) |
| Fear of stigmatization (social disapproval) of myself | 0 (0) | 0 (0) | 0 (0) | 0 (0) |
| Its form/mode of administration (e.g., dried herb/resin) is poorly adapted to certain patients | 0 (0) | 3 (1.7) | - | - |
